# Supplementary material for: Causal Relationship between Aging and Anorexia Nervosa: A White-Matter-Microstructure-Mediated Mendelian Randomization Analysis
Source: Biomedicines. 2024 Aug 16;12(8):1874. doi: 10.3390/biomedicines12081874 (PMC11351342; doi:10.3390/biomedicines12081874)
Supplement: Supplementary file 1 [file biomedicines-12-01874-s001.zip › Supplementary Figure/Figure S4/Singe SNP plot of MR analysis for TL to ubm-b-1816.pdf]

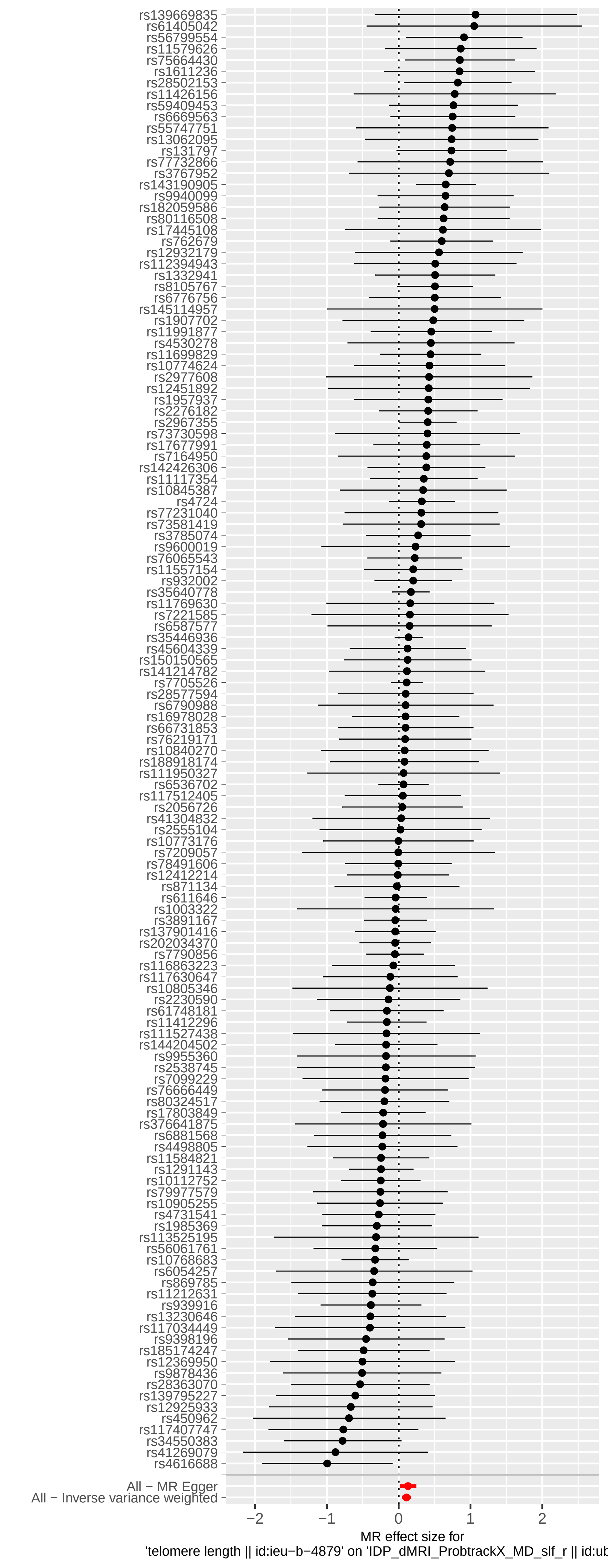
rs139669835
rs61405042
rs56799554
rs11579626
rs75664430
rs1611236
rs28502153
rs11426156
rs59409453
rs6669563
rs55747751
rs13062095
rs131797
rs77732866
rs3767952
rs143190905
rs9940099
rs182059586
rs80116508
rs17445108
rs762679
rs12932179
rs112394943
rs1332941
rs8105767
rs6776756
rs145114957
rs1907702
rs11991877
rs4530278
rs11699829
rs10774624
rs2977608
rs12451892
rs1957937
rs2276182
rs2967355
rs73730598
rs17677991
rs7164950
rs142426306
rs11117354
rs10845387
rs4724
rs77231040
rs73581419
rs3785074
rs9600019
rs76065543
rs11557154
rs932002
rs35640778
rs11769630
rs7221585
rs6587577
rs35446936
rs45604339
rs150150565
rs141214782
rs7705526
rs28577594
rs6790988
rs16978028
rs66731853
rs76219171
rs10840270
rs188918174
rs111950327
rs6536702
rs117512405
rs2056726
rs41304832
rs2555104
rs10773176
rs7209057
rs78491606
rs12412214
rs871134
rs611646
rs1003322
rs3891167
rs137901416
rs202034370
rs7790856
rs116863223
rs117630647
rs10805346
rs2230590
rs61748181
rs11412296
rs111527438
rs144204502
rs9955360
rs2538745
rs7099229
rs76666449
rs80324517
rs17803849
rs376641875
rs6881568
rs4498805
rs11584821
rs1291143
rs10112752
rs79977579
rs10905255
rs4731541
rs1985369
rs113525195
rs56061761
rs10768683
rs6054257
rs869785
rs11212631
rs939916
rs13230646
rs117034449
rs9398196
rs185174247
rs12369950
rs9878436
rs28363070
rs139795227
rs12925933
rs450962
rs117407747
rs34550383
rs41269079
rs4616688
All − MR Egger
All − Inverse variance weighted
−2
−1
0
1
2
MR effect size for
'telomere length || id:ieu−b−4879' on 'IDP_dMRI_ProbtrackX_MD_slf_r || id:ub
